# Experiences of women participating in a human papillomavirus-based screen-triage-and treat strategy for cervical cancer prevention in Malawi

**DOI:** 10.3389/fonc.2024.1356654

**Published:** 2024-02-27

**Authors:** Fan Lee, Shannon McGue, John Chapola, Wezzie Dunda, Jennifer H. Tang, Margret Ndovie, Lizzie Msowoya, Victor Mwapasa, Jennifer S. Smith, Lameck Chinula

**Affiliations:** ^1^ Department of Obstetrics and Gynecology, University of North Carolina, Chapel Hill, NC, United States; ^2^ Department of Medicine, Duke University, Durham, NC, United States; ^3^ Department of Health Policy and Management, Gillings School of Global Public Health, University of North Carolina, Chapel Hill, NC, United States; ^4^ University of North Carolina Project-Malawi, Lilongwe, Malawi; ^5^ Lineberger Comprehensive Cancer Center, University of North Carolina, Chapel Hill, NC, United States; ^6^ Department of Epidemiology and Biostatistics, Kamuzu University of Health Sciences, Blantyre, Malawi; ^7^ Department of Epidemiology, University of North Carolina Gillings School of Global Public Health, Chapel Hill, NC, United States

**Keywords:** HPV self-collection, VIA triage, thermal ablation, cervical cancer screening experience, Malawi (MeSH [Z01.058.290.175.500])

## Abstract

**Objective:**

To explore the experiences of Malawian women who underwent a human papillomavirus (HPV)-based screen-triage-treat algorithm for cervical cancer (CxCa) prevention. This algorithm included GeneXpert® HPV testing of self-collected vaginal samples, visual inspection with acetic acid (VIA) and colposcopy for HPV-positive women, and thermal ablation of ablation-eligible women.

**Method:**

In-depth interviews were conducted with participants of a trial that evaluated the feasibility of a HPV-based screen-triage-treat algorithm among women living with HIV and HIV negative women in Lilongwe, Malawi. Participants were recruited from 3 groups: 1) HPV-negative; 2) HPV-positive/VIA-negative; 3) HPV-positive/VIA-positive and received thermal ablation. Interviews explored baseline knowledge of CxCa and screening, attitudes towards self-collection, and understanding of test results. Content analysis was conducted using NVIVO v12.

**Results:**

Thematic saturation was reached at 25 interviews. Advantages of HPV self-collection to participants were convenience of sampling, same-day HPV results and availability of same-day treatment. There was confusion surrounding HPV-positive/VIA-negative results, as some participants still felt treatment was needed. Counseling, and in particular anticipatory guidance, was key in helping participants understand complex screening procedures and results. Overall, participants expressed confidence in the HPV screen-triage-treat strategy.

**Discussion:**

HPV testing through self-collected samples is a promising tool to increase CxCa screening coverage. A multi-step screening algorithm utilizing HPV self-testing, VIA triage and thermal ablation treatment requires proper counseling and anticipatory guidance to improve patient understanding. Incorporating thorough counseling in CxCa screening programs can change women’s perspectives about screening, build trust in healthcare systems, and influence healthcare seeking behavior towards routine screening and prevention.

## Introduction

Malawi has the highest cervical cancer (CxCa) mortality rate in the world (51.5 deaths/100,000 per year), seven times higher than the global rate (1). This disease burden is largely due to the high prevalence of HIV (>9% for women 15-50 years of age) (2) and low CxCa screening coverage (3). For the last two decades, the national CxCa screening program in Malawi has been using visual inspection with acetic acid (VIA) for screening and cryotherapy for treatment of VIA-positive lesions amenable for ablative therapy. However, a comprehensive evaluation of this program in 2015 showed that screening coverage has remained low (<27%) and less than half of those who required treatment received treatment (3).

Lack of trained staff was cited as the main challenge in offering CxCa screening by service providers in Malawi (4). Pelvic exam-based screening for cervical cancer, such as VIA, requires trained providers, adequate facilities, and patient acceptability of exams, thus limiting screening efficiency, access, and uptake. Human papillomavirus (HPV) screening by self-collection can bypass some of these challenges. The detection of high-risk types of HPV associated with CxCa has improved the accuracy of detecting cervical precancer (5) and was recommended by the World Health Organization (WHO) in 2021 to be the primary screening method, when available (6). Recent advancements in technology, such as GeneXpert^®^ HPV tests (Cepheid Inc, Sunnyvale, CA, USA), has made HPV testing available in low-resource settings and allow the possibility of same-day treatment because of the quick result turnaround (about 1-2 hours with GeneXpert^®^). Self-collection of vaginal sample for HPV testing has been validated as an effective and sensitive method for CxCa screening if highly sensitive assays are utilized, with notably lower provider burden compared to provider-collected tests (5). HPV self-collection has been shown to increase CxCa screening compared to VIA (7) and is acceptable to women all over the world (8), including in Sub-Saharan Africa (SSA) (9).

The biggest limitation to same-day treatment of cervical precancerous lesions in Malawi was maintaining functional cryotherapy machines and sustaining the supply of refrigerant gas (3). Thermal ablation, a battery-powered, portable, and less time-intensive treatment modality is replacing cryotherapy as the preferred treatment modality in low- and middle-income countries (LMICs). With increasing evidence of safety, efficacy, and the ability to increase same-day screening and treatment (10, 11), the Malawi Ministry of Health (MoH) added thermal ablation as a treatment option in the national screening guidelines (12).

In line with WHO and Malawi MoH recommendations, we implemented a HPV-based screen-triage-treat algorithm that incorporates the strategies of HPV self-collection and same-day thermal ablation treatment. Our single-arm prospective trial evaluated the feasibility and performance of this algorithm in Lilongwe, Malawi among women living with HIV (WHIV) and HIV-negative women (13). This manuscript focuses on the study’s secondary aim, which was to explore the experiences of the participants. Perceptions of HPV-based screening have primarily been evaluated in HPV/Pap smear algorithms among patients in high income countries (14, 15). This study uniquely evaluated the experience of women undergoing a HPV/VIA screen-triage-treat algorithm, including acceptance of HPV self-collection, understanding of results, and challenges posed by a multistep screening process.

## Methods

### Study setting and participants

Participants for this qualitative sub-study were recruited from a single-arm prospective study that investigated a novel HPV screen-triage-treat strategy among 1,250 women (625 WHIV and 625 HIV-uninfected) in Lilongwe, Malawi. The strategy consisted of 1) GeneXpert^®^ HPV testing of self-collected cervicovaginal samples; 2) VIA and colposcopy for HPV-positive women; and 3) thermal ablation for HPV-positive/ablation-eligible women. Colposcopy was conducted following VIA to determine final eligibility for thermal ablation, and additional samples (endocervical curettage and cervical biopsy or pap smear) were collected based on colposcopy results for other study objectives (see protocol paper for details) (13). For this qualitative sub-study, we focused on the HPV self-collection screening, VIA triage, and thermal ablation treatment components of the algorithm.

The parent study and this qualitative sub-study were conducted at UNC Project-Malawi’s Tidziwe Centre clinic in Lilongwe, Malawi. UNC Project-Malawi is a collaboration between the University of North Carolina at Chapel Hill and the Malawi MoH. Recruitment of participants for the parent study are detailed in the protocol paper (13). Briefly, study staff provided educational talks about cervical cancer screening and the study in waiting areas of outpatient clinics that provided reproductive health and HIV care services. Women who were interested in the study were scheduled for screening at the UNC Project-Malawi research clinic. On arrival, informed consent began with a summary of study goals and introduction to the new approach to cervical cancer screening employed in the study. Specifically, counseling was focused on HPV, its relationship to cervical cancer and detailed descriptions of each screening step. For those who continued to be interested, the remainder of the informed consent was completed, which further included counseling on what to expect at each step of screening and after each result. During screening, participants’ understanding of procedures and results was continually assessed and counseling was reiterated as needed. Study eligibility criteria included: femal1es between 25-50 years of age; non-pregnant; at least 12 weeks postpartum; and able and willing to provide written informed consent. Exclusion criteria included: current or prior history of cervical, vaginal, or vulvar cancer; current symptomatic sexually transmitted infection (STI) requiring treatment; prior HPV vaccination; allergy to acetic acid; or history of total hysterectomy.

At the enrollment visit of the parent study, participants were asked if they were interested in joining the qualitative sub-study. Convenience sampling was used among those who expressed interest and the sub-study participants were recruited via phone call by study staff. Those interested were asked to come back to Tidziwe Centre to consent and enroll in the sub-study. We planned to enroll up to thirty women for in-depth-interviews (IDIs) across 3 groups of screening outcomes: 1) those who screened negative on self-collection HPV test (HPV-negative); 2) those who screened positive on self-collection HPV test but had a negative VIA (HPV-positive/VIA-negative) and did not receive treatment; and 3) those who screened positive on HPV self-collection, had a positive VIA triage exam (HPV-positive/VIA-positive) and received same-day thermal ablation. All participants received transport reimbursement, as approved by the local ethics committee.

### Data collection

Semi-structured IDIs were developed around four main domains of inquiry: 1) baseline knowledge and perception of CxCa and CxCa screening; 2) attitudes towards self-collection; 3) experience with the screen-triage-treat procedures; and 4) understanding of screening results. Domains were developed based on existing literature that assessed acceptability of and perspectives on HPV-based screening (9, 16, 17). Interviews were conducted in Chichewa, the local language, by study staff experienced in qualitative data collection methods (WD). Interviews were audiotaped and then translated and transcribed into English. Completed transcripts were reviewed immediately by study investigators and analyzed for emerging and/or new themes to inform the questions for subsequent interviews. This iterative process ensured saturation of themes and depth of content within the predetermined domains of inquiry.

Additionally, five survey questions specific to participants’ HPV self-collection experience were administered after IDIs. The survey asked participants to rank whether they felt embarrassed, experienced discomfort, experienced pain, had privacy, and felt confident that they self-collected correctly on a three-point scale of 1) not at all; 2) somewhat; and 3) very much.

### Data analysis

Three qualitative investigators (FL, SM, JC) independently reviewed and coded three transcripts at a time using thematic analysis, followed by a group discussion, to identify relationships between emerging themes and ensure relevance to research questions. This was repeated until the code book was finalized. The code book and IDIs were uploaded to NVIVO 12 software. To ensure validity of coding and robustness of analysis, nine interviews were coded by all three investigators and the remaining 16 were doubly coded. The code book was refined as analysis progressed. An Excel spreadsheet was used to structure and compile all extracted quotes for each code. The quotes were reviewed by all three coders and themes were revised until it was felt that they accurately reflected the data.

Survey data on HPV self-collection experience was entered into an Excel spreadsheet, and descriptive statistics were used for analysis.

### Ethical considerations

This study was approved by Malawi National Health Sciences Research Committee and the University of North Carolina at Chapel Hill Institution Review Board. All participants underwent informed consent and provided written consent.

## Results

**Figure 1:**
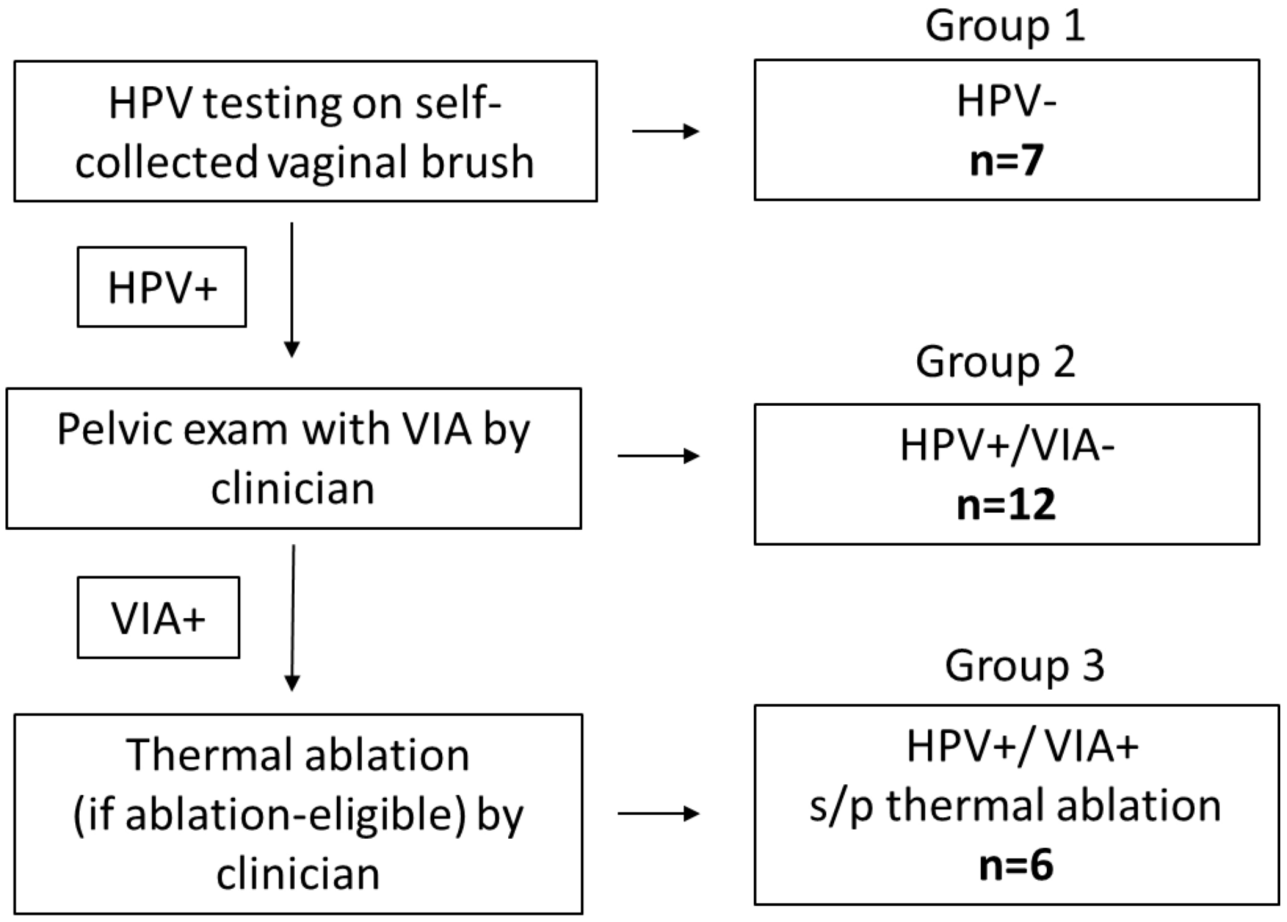
Screening algorithm, screening outcomes (Group 1: HPV-negative; Group 2: HPV-positive/VIA-negative; Group 3: HPV-positive/VIA-positive and underwent thermal ablation) and number of participants interviewed in each group. HPV, Human papillomavirus (testing for high risk HPV); VIA, Visual inspection with acetic acid s/p: status post. Healthcare provider included nurses and clinicians.

Between July 2020 – March 2021, we interviewed 25 women across the three groups of screening outcomes. Thematic saturation was reached at seven for Group 1, twelve for Group 2, and six for Group 3 (**Figure 1**).

### Baseline characteristics of participants

Participants were between 25-49 years old, with a median age of 34 (**Table 1**). Overall, ten (40%) of participants were WHIV (one in Group 1, five in Group 2, and four in Group 3). Most participants (84%) reported no prior CxCa screening; for those who did, the prior screening method reported was VIA. The majority of participants (72%) report a travel time between 30-60 minutes to reach the nearest clinic that offered CxCa screening. Of note, participants in Group 3 had a longer time between initial HPV screening and their IDI (median of 7 weeks vs. 3-4 weeks for Groups 1 & 2). Baseline socioeconomic characteristics were similar across the three groups. Most (60%) had at most some secondary school education, most (68%) were married or living with their partner, and most (64%) worked outside the house. Half of the participants (48%) made less than the equivalent of about $2 per day (<K49,999 per month), and the others (52%) made the equivalent of about $2-6 per day (K50,000-K150,000 per month). The median age at first vaginal intercourse among participants was 17 years, with a range of 15-22 years. The median number of lifetime partners was two, with a range of 1-3. Most (76%) reported using condoms at least some of the time.

**Table 1:** Baseline demographic of participants by self-collection HPV screening and VIA triage result.

Characteristics	Total N=25 n(%)	Group 1 HPV- n=7 n(%)	Group 2 HPV+ / VIA- n=12 n(%)	Group 3 HPV+ / VIA+ n=6 n(%)
Age median (range) in years	34 (25-49)	36 (27-49)	31 (26-40)	28 (25-36)
HIV positive*	10 (40%)	1 (14%)	5 (45%)	4 (57%)
No prior cervical cancer screening^†^	21 (84%)	6 (86%)	9 (75%)	6 (100%)
Median length of time (range) in weeks between screening and in-depth-interview	8 (1.3-24)	3.6 (1.3-9.1)	4.7 (2.0-24.0)	7.1 (3.4-17.1)
Educational attainment^‡^			–	
Some secondary or less	15 (60%)	5 (71%)	4 (33%)	6 (100%)
Completed secondary or more	8 (32%)	1 (14%)	7 (58%)	0
Marital Status				
Single (never married, widowed, divorced or separated)	8 (32%)	1 (14%)	4 (33%)	3 (50%)
Married or living with partner	17 (68%)	6 (86%)	8 (67%)	3 (50%)
Monthly income^§^				
Less than $65 (K49,999)	12 (48%)	3 (43%)	6 (50%)	3 (50%)
$65-$195 (K50,000-K150,000)	13 (52%)	4 (57%)	6 (50%)	3 (50%)
Distance to nearest clinic that offers cervical cancer screening services (CCS)				
<30 min	14 (56%)	4 (57%)	6 (50%)	4 (67%)
30-60 min	11 (44%)	3 (43%)	6 (50%)	2 (33%)
Parity median (range)	3 (0-5)	3 (2-4)	2 (0-5)	3 (1-4)
Age at first vaginal intercourse: median (range)	17 (15-22)	19 (15-20)	17 (16-22)	15 (15-21)
Number of lifetime sexual partners: median (range)	2 (1-3)	2 (1-2)	1 (1-3)	2 (2-2)
Uses condoms	19 (76%)	7 (100%)	9 (75%)	6 (100%)

* All WHIV are on ART and have been taking it for over 6 months.

^†^ All 4 participants with prior screening had VIA screening.

^‡^ No response = 2.

^§^ Malawi Kwacha(K) to United States Dollar ($) exchange at the time of study was 1K to $0.0013; K49,999= ~$65; K150,000 = ~$195.

**Participants reported between 1-4 family planning methods used, most commonly used was Depo provera IM (n=16), followed by Jadelle (n=8), Depo provera SC (n=6), Levoplant (n=4), birth control pills (n=4), and Implanon (n=2).

### Knowledge and perception of CxCa

All participants had heard of CxCa, predominantly from CxCa screening messages on the radio, at clinics (antenatal, family planning and HIV), and/or from other women in the community (**Table 2**). Participants’ reported that CxCa is associated with sexual transmission, early sexual debut, and having multiple sexual partners, specifically with uncircumcised men. Some participants described CxCa as asymptomatic in the early stage. Others described gynecological symptoms, such as vaginal discharge, abnormal vaginal bleeding, abdominal pains, and genital sores, as associated with CxCa. Several participants reported that CxCa can spread to the womb and treatment involves hysterectomy, which leads to the inability to give birth again. The general perception of CxCa was that it is dangerous and deadly when detected in advanced stages when it is too late for treatment (**Table 2**). All felt that CxCa can be prevented; some reported prevention through lifestyle changes, such as limiting number of sexual partners, male circumcision, and vaginal hygiene. Several participants also specifically reported that CxCa can be prevented with screening.

**Table 2:** Participants’ prior knowledge and perception of cervical cancer and cervical cancer screening before undergoing self-collection HPV-based screen-triage-and treat program.

Area of inquiry	Themes	Quotes
Participants’ prior knowledge of cervical cancer	Heard about cervical cancer screening	I didn't know anything [about cervical cancer]. What cancer looks like, or what happens. I would just hear that people are getting screened for cancer. (P0003)
	Cervical cancer is sexually transmitted, transmitted from uncircumcised men and the risk is increased with having multiple sexual partners	If you have unprotected sex whilst still young, you are bound to contract HPV. They [family planning clinic] also said that an uncircumcised man harbors HPV, because the virus thrives in warm places. They further went on to say all men have HPV, they have it from a young age, they are born with it. So if young men have sex with young women, they transmit the virus easily. (P0685)
	Gynecologic symptoms are associated with cervical cancer	I heard that when you have cervical cancer, you experience abdominal pains, and you also develop sores. Likewise, your vaginal discharge has a foul smell, and is watery with a yellowish color. (P0007)
	Treatment associated with hysterectomy and infertility	[At antenatal clinic] they would just say that there is cervical cancer, and if it spreads, a hysterectomy is performed, and a person will never be able to give birth again after that. (P0322)
Participants’ perception of cervical cancer	Cervical cancer is perceived as dangerous and deadly	I knew that cervical cancer is very dangerous...They said if you have cervical cancer, it is only detected when it’s too late. (P0131)
	Asymptomatic in early stages	With cancer, you do not feel any pain, as opposed to malaria where you feel body pains. That is why I made a decision to get screened for cancer so I know what my health is like. You can just wake up one day and you have cancer. (P0310)
	Cervical cancer can be prevented	When you go to get screened and you hear your results, if the cancer is in its early stages, it is preventable (P0003)
Participants’ prior knowledge of cervical cancer screening	Community stigma of screening: fear of speculum exams	I knew that cervical cancer is very dangerous...They said if you have cervical cancer, it is only detected when it’s too late, due to the method that we have in Malawi, the one where a metal is placed on the opening of the vagina. A lot of women we are afraid of this. So we only know when we are sick, that it is cancer.(P0131)
	Many understood that screening was a key part of cervical cancer prevention	You can prevent it [cervical cancer] by getting screened at the hospital, hearing the results, and following what the doctors tell you. (P0684)
	Heard about screening at health facilities, radio, community and friends	I just heard people talking about it, even in hospitals, in here about it. (P0687)
Participants’ perception of cervical cancer screening	Decision to get screening overall despite fear of screening	Made the decision to get screened so that I know about my health where cervical cancer is concerned. However, I was afraid to go and get screened because women would often talk about how painful it is, and would get me scared.(P0478)
	Screening can help catch abnormalities early when treatment may still be available.	I know that if we get screened for cervical cancer then we know about our health. Whether we have cervical cancer or not. If we have it, then we have to follow procedures so that the disease can be cured. If we don’t have, then we ought to go and take care of ourselves so that we don’t get it. (P0112)

### Perceptions about CxCa screening

Participants reported that in their communities, screening is associated with the embarrassment and painful speculum exams (**Table 2**) and that these fears hinder screening attendance. Many had heard about screening through clinics (e.g., antenatal or family planning), and some were encouraged by clinicians or other women to attend screening. Some expressed initial hesitancy towards screening based on hearsay from the community, however, all participants had a positive perception of screening and believed that screening can prevent disease. The predominant reason participants decided to undergo screening was to know about their health and to catch abnormalities at an early stage when treatment was still available.

### Experience of self-collection

**Figure 2:**
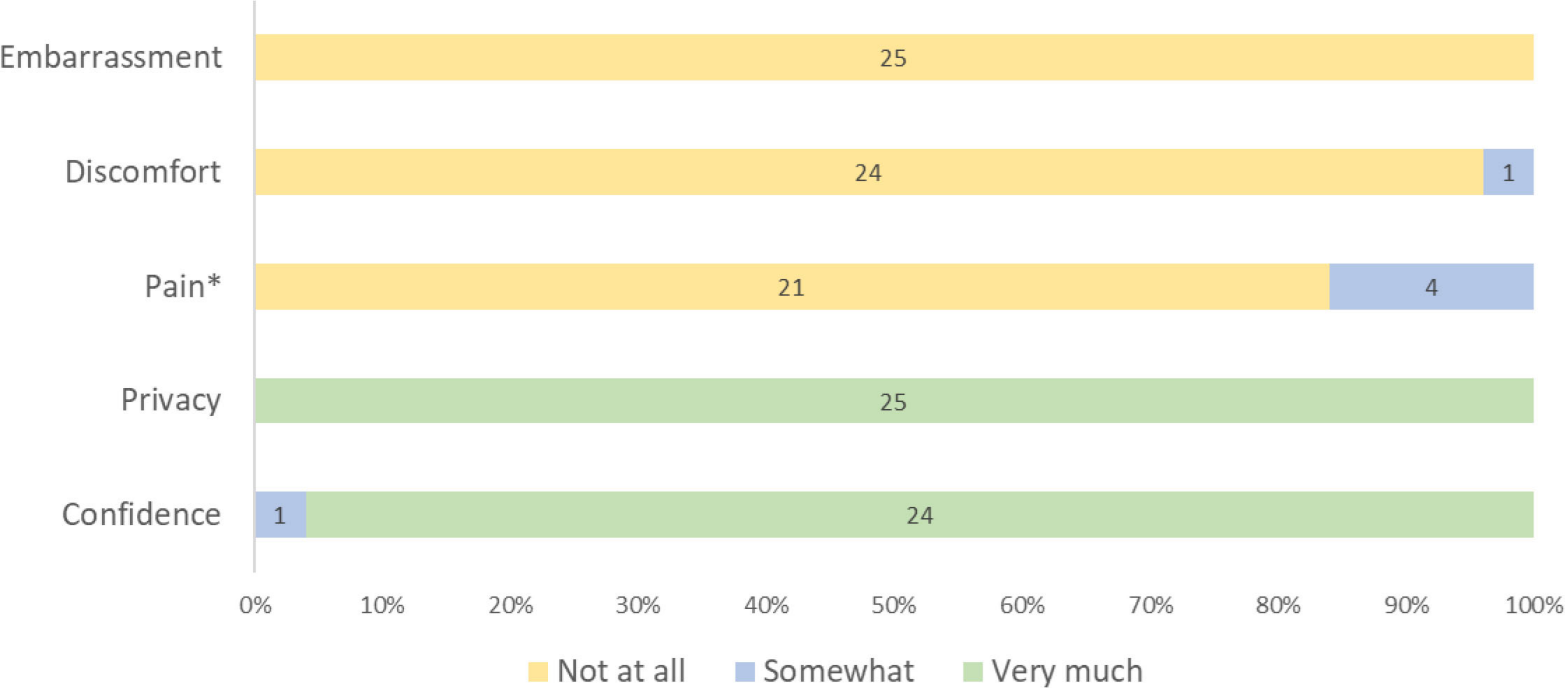
Participants’ reported HPV self-collection experience on a three point scale (N=25). ^*^ Of those who reported “somewhat” pain during HPV self-collection, 3 had HPV negative and 1 had HPV positive results.

All participants reported a positive overall experience with self-collection of vaginal samples for HPV testing (**Table 3**). All expressed understanding of the self-collection process and many were able to describe the steps in detail even weeks after screening. They felt well-counseled on the collection steps, described it as an easy procedure, and valued the quick turn-around of HPV results. When participants were asked to rate a series of experiences during self-collection, all 25 participants reported no embarrassment, only one reported discomfort (rated as mild), and only four reported pain (rated as mild by all four) (**Figure 2**). On further inquiry, mild pain was described as more of a “discomfort” or “sensation” or “pinch” by participants (**Table 3**). All participants reported they had very good privacy, and all but one reported being very confident in collecting the sample correctly (**Figure 2**). Many suggested that this method could be more acceptable to women who feel embarrassed to undress for speculum-based screening (**Table 3**).

**Table 3:** Participants’ experience with self-collection HPV-based screen-triage-and treat program.

Area of inquiry	Themes	Sub-themes	Quotes
HPV self-collection	Participants reported being well counseled, which was reflected in their ability to remember steps of self-collection well, and felt that avoiding pelvic exams was a strength of self-collection.	Remembered the steps of self-collection well	When I went in a private room to self-collect... I inserted the brush slowly, then I felt that I had reached the cervix, and I swirled the brush 4 times and pulled it out. I looked at the brush if the sample was visible, I saw it, I then put the brush in the tube (without touching it). After that, I handed it over. (P0478) [*Participant was interviewed 8.4 weeks after screening]*
		Valued counseling about screening process	What I liked most was they started by taking us through what happens. So we knew what happens, before it took place. This was so we should make a personal decision whether to go ahead or not.. (P0409)
		Valued doing the collection herself and quick turn-around of HPV results after collection	I liked that I collected the brush, and I got my results after the brush had been tested. (P0467)
		Avoiding pelvic exams decreases fear, is more private, and can reach more women	I was also one of the people who would be embarrassed with the other method, where there would be undressing, and male doctors would see that nakedness. A lot of people do not like that. (P0322)
	Most initial concerns about self-collection resolved after counseling and/or performing the self-collection	Worried about treatment after a positive result	I was worried because I thought I will be found with a problem, which will result in a hysterectomy on my part. I was very worried that if they remove my womb, I will never give birth again. (P0131)
		Worried it might be painful or uncomfortable, relieved by seeing/touching the brush	Before I collected the swab, my worry was whether the brush would really be inserted and if I would feel pain, and also if the brush would wound me. But when I touched it, I concluded that it wasn’t the case. When I inserted it into my vagina, I realized there was no pain and everything they told me to do was possible. (P0007)
		Worried about collecting sufficient sample.	Yes, I had worries. I was told that if the sample will be insufficient on the brush, then I would have to repeat the process. So I was worried about that. So I made sure I did it right the first time. (P0118)
Visual Inspection with Acetic Acid (VIA) triage	Despite fear and discomfort of pelvic exam performed for VIA triage, most felt comfortable due to the counseling they received throughout the exam process	Discomfort during the pelvic exam was mostly due to speculum (insertion of the metal)	I was scared when I saw the speculum…But I was well assisted, and I was being told what was happening the whole time... I felt a bit of pain, but I think that was caused by a mishap…But when the speculum was inside and they were doing the exam, I didn’t feel anything, I was just conscious that there is a foreign body inside me. Only when they were taking out the speculum did I feel a bit of pain. (P0322)
		Pelvic exam was embarrassing, especially with male provider	Embarrassment is very likely being a male [doctor] was present. (P0310)
		Did not like additional test following HPV positive self-test result	I didn’t like when the doctors told me I needed to go for another test. (P0682)
		Active counseling during the exam was helpful	They were telling me everything that was happening. Some don’t tell you what is happening, you just realize it has happened. That is very heartbreaking, but here, it was a delight to be told what was happening. And they addressed us with warmth.(P0322)
Thermal ablation treatment	Some women reported initial anxiety about the treatment, others had mild discomfort during the treatment. But overall, thermal ablation was painless and well tolerated. Many participants expressed gratitude for the treatment.	Some had anxiety about the treatment procedure	When they told me that they were going to perform thermal ablation, I was anxious. The hot air got me worried. (Chuckles), but I accepted the situation and went on ahead. (P0629)
		Some experienced mild pain but no severe discomfort	It was a bit painful… When they finished, they gave me cotton wool to use. I got off the bed with no problems. I was able to walk, I got home just fine, I did not encounter any problems further than that. (P0118)
		Grateful for availability of quick treatment following initial screening	The treatment was instantaneous. From the pelvic exam to the thermal ablation. And then I went back home. There was no postponement of anything. I really liked that (P0629)
Post-screening and treatment experience	Post-treatment symptoms were minor and mostly consisted of vaginal discharge	Several described vaginal discharge after the procedure that stopped spontaneously, most were not alarmed as they were counseled that this may be a side effect of treatment.	The complication I experiences happened when I got home, but they had already warned me about it. They told me that I’d produce dark vaginal discharge. It seemed like a menstrual flow, but it was very dark. That went on for a week. But after it stopped, it never happened again until now. (P0467)
	The post-treatment abstinence recommendation* was difficult for participants. *Post-procedural abstinence counseling changed from 6 to 4 weeks over the interview period.	The need to discuss abstinence with male partner caused anxiety	I kept thinking the whole way back home, about how I was going to explain this [abstinence] to my husband. Because as it was, I wasn’t given any medication for me to show him that maybe he can believe me. It was only I who knew of how I had been assisted, and what to do afterwards. So for me to go and tell him about the 6 weeks. It was a problem." (P0131)
		Many were not able to abstain from sex due to lack of partner support	They just told me not to indulge in sexual intercourse in the time being. Should it happen that I really want, I should use protection. But because my husband was away, I managed to stay without sexual intercourse for 3 weeks. (P0310)
		Desired male partner or couples counseling	It was better that I be sent back home and come back together on a day that he [my husband] can manage to come…To screen and treat me, then explain to both of us of what is to happen. Then after, to stay away from each other sexually for such and such a long time. Explain everything, just as they did to me. (P0131)
	Participants were excited to their experiences with friends and family	Sharing experiences address misconceptions and encourages screening uptake by friends	I told my friends, almost 10 of them if I’m not mistaken. I told them to go and get screened for cervical cancer… They asked if it was scary. I explained to them the process I went through, and the method. I explained to them that I was given a brush, you self-collect, and it is not painful when it is inserted to collect the sample. (P0578)

### Understanding HPV results

All participants, except one, reported that they had never heard of HPV prior to being involved in the research study. After undergoing screening and counseling, participants were able to describe HPV as a virus that can cause CxCa or lesions on the cervix (**Table 4**). Regardless of their own HPV result, participants reported that a HPV-positive test is associated with increased risk of CxCa, which requires further evaluation and/or treatment. Participants also recognized that while HPV-negative result was reassuring, regular screening is still necessary since the infection could occur later. The concept of routine screening was compared to HIV testing by both WHIV and HIV-negative women.

**Table 4:** Participants’ overall impression and major take away messages from the self-collection HPV-based screen-triage-and treat strategy.

Theme	Sub-theme	Quotes
Thorough counseling contributed to their positive overall experience	Thorough counseling minimizes negative emotions, built trust with the providers and leads to satisfaction with the screening and treatment services	I understood what they were trying to say. I was not afraid when they explained to me. Everything that I felt, fear and doubt, it all went away. I was relieved. I went back home happy. I realized that if I had just stayed at home and not gotten screened, it could have been disastrous. But this time around, everything was fine. (P0478)
	Anticipatory guidance helps participants know what to expect to limit the confusion of a multi-step or multi-result screening method	What I liked most was they started by taking us through what happens. So we knew what happens, before it took place. This was so we should make a personal decision whether to go ahead or not. The second thing was when we heard that, it was good because we were told everything beforehand. Yes, that’s what we encountered on that day. (P0409)
	Ongoing active counseling throughout exams and treatment procedures is reassuring for participants	They were telling me everything that was happening. Some don’t tell you what is happening, you just realize it has happened. That is very heartbreaking, but here, it was a delight to be told what was happening. And they addressed us with warmth. (P0322)
	Counseling extended to health maintenance and routine screening for prevention	I was treated well; I was told what I needed to hear. They told me there is no problem at the moment, but it could develop in the future. So we needed to do such and such. (P0068
Participants felt it was important to share their screening experiences, addresses misconceptions and encourages screening	Many participants already shared their experience with others (family and friends) in the community, addressed misconceptions and encouraged other women to go for screening	First, [I told] my husband. Second, my friends. I was encouraging them to go and get screened for cervical cancer….I was the one encouraging them due to the questions they were asking me…I was explaining to them that you self-collect the sample…Don’t be afraid of a pelvic exam. It is better here because of the self-sample collection. I was encouraging them in that manner. (P0112)
	Sharing their screening and treatment experiences was important	Also that a lot of us women are usually hesitant to come to the hospital, but that is not good. If you’re summoned to come to the hospital, you should do just that. You only have one life. If you lose it, there’s no getting it back. So personally, I just want to encourage women to take part in this, because it is a good thing. And it is simple. (P0004)
	Many participants themselves came for screening due to hearing about it from other women in the community	I’d just hear about how some women had gone for cervical cancer screening. These women testifying about their experiences is what prompted me to come as well (P0467)

### Experience of VIA triage and thermal ablation treatment

Several participants who underwent VIA triage (Groups 2 and 3) reported negative experiences during pelvic exams (**Table 3**). Some reported discomfort related to the insertion and removal of the speculum, and others reported feeling embarrassed, especially when male providers were present. One participant did not like having to go for another test after the initial HPV self-collection. The discomfort and embarrassment were ameliorated by ongoing counseling during the exam process (**Table 5**). When thermal ablation was recommended, some participants reported having anxiety about the treatment procedure and others reported mild discomfort during treatment. However, overall, most expressed that thermal ablation itself was quick, painless, and well tolerated. Many participants who underwent treatment expressed gratitude for the treatment.

**Table 5:** Participants’ understanding of results and purpose of screening, triage and treatment steps stratified by HPV result.

Area of Inquiry	Themes	HPV- participants Quotes	HPV+ participants Quotes
Understanding HPV in the context of cervical cancer	HPV is a virus; HPV causes cervical cancer; HPV causes lesions^*^ on the cervix. No major differences in understanding between HIV-positive or HIV-negative participants	It [HPV positive test] means she has a virus that causes cervical cancer. So that means she has to receive treatment at the hospital in order to kill the viruses so that they don’t cause cervical cancer. (P0003) *HPV negative, HIV negative*	They told me that testing HPV positive does not directly mean that you have cervical cancer. It is merely the virus that causes cervical cancer, but if the virus has caused lesions on the cervix, then you likely have cervical cancer. (P0685) *HPV positive, VIA negative, HIV positive*
Understanding HPV results in the context of cervical cancer	HPV negative result does not need treatment, but still needs routine screening because there is still risk in the future	Not that I am above others, but at this particular time, it meant that I am okay. However, this does not mean it is forever, maybe at some point I may be diagnosed with it. But I am okay for now. (P0490) *HPV negative, HIV negative*	That [HPV negative] means that woman does not have cervical cancer….Yes, she needs further testing after some time… No. She does not need any treatment. Only if she was later on tested and the test results, positive. Only then. (P0409) *HPV positive, VIA negative, HIV positive*
	HPV positive result is associated with risk and needs follow-up and/or treatment	In my understanding, when that [HPV positive result] happens, then she is supposed to be screened again, this time around, a pelvic exam...And then she undergoes thermal coagulation. (P0007) *HPV negative, HIV negative*	It [HPV positive result] meant that chances of me getting cancer in the future were high. (P0068) *HPV positive, VIA negative, HIV negative*
Understanding implication of HPV result in the context of cervical cancer prevention	Continued routine periodic screening is necessary for prevention, even if HPV screening is negative	Maybe the virus may come back, but I should not stop getting screened, so that I know the status of my health. (P0628) *HPV negative. HIV negative*	Yes [she needs further testing if she is HPV negative ] Because even though at that particular time she tested negative, she can test positive at a later date. (P0467) *HPV positive, VIA positive, HIV positive*)
	Some compared routine cervical cancer screening to HIV screening. No major differences between HIV positive versus negative participants	You are supposed to get tested for HIV every 3 months. At this moment I am fine, but how about 3 or 4 months down the line…I find it to be a good thing to get screened (for cervical cancer) again. (P0490) *HPV negative, HIV negative*	It is like HIV, you cannot get tested once and think you are in the clear. (P0684) *HPV positive, VIA negative, HIV negative*
Understanding the role of VIA in this screening algorithm	VIA is to examine the cervix , to see if there is damage caused by the virus. VIA detects lesions that would require treatment. VIA is a confirmatory test.	So that they confirm if I have the disease or not. (P0682) *HPV negative. HIV negative*	They told me I had tested HPV positive and needed a pelvic exam so that they check my cervix, to see if there it had lesions. (P0409) *HPV positive, VIA positive, HIV positive*
Area of inquiry	Themes	Quotes: HPV+ participants	
Understanding HPV+/VIA- (positive HPV-screen, negative VIA triage) result	A positive screening (HPV) followed by negative triage (VIA) and therefore no treatment was confusing for some	I am worried that one day if I don’t get screened again, maybe the HPV will develop again. (P0478) *HPV positive, VIA negative, HIV positive* I was a bit worried, because I was HPV positive, and then I was told I am VIA negative. I was a bit confused, but I accepted it. After they said that in the future, a problem can develop and we ought to fix now what we can, I agreed to that, before anything gets bad. I accepted that. (P0068) *HPV positive, VIA negative, HIV negative*	
	Some participants felt they still needed some kind of treatment, another thought they were treated	*This participant expressed need for treatment despite VIA negative exam:* The treatment in the sense that I am worried that HPV may reoccur on the cervix. And if so, how can it be managed? (P0684) *HPV positive, VIA negative. HIV negative.* They told me I do not have any lesions, so they are going to smear vinegar so as to kill the HPV. (P0685) *HPV positive, VIA negative. HIV negative*	
Understanding of HPV+/VIA+ result (positive HPV-screen, positive VIA triage) and need for thermal ablation treatment	Understood that a positive VIA meant treatment was indicated	I was told that I have lesions that could lead to cervical cancer. I was also told not to worry and that I would be treated the same day, and I was. (P0131) *HPV positive, VIA positive, HIV negative* I accepted it, because I got treatment for it, as opposed if I had just neglected the situation, it could have been disastrous. But it was a good thing that I came to the hospital and I was assisted. (P0467) *HPV positive, VIA positive. HIV positive*	
Understanding the purpose of thermocoagulation	Thermal ablation was understood to treat lesions and prevent worsening of disease *No major differences in answers between HIV pos and HIV neg participants	To treat the lesions so that the situation doesn’t get worse. (P0467) *HPV positive, VIA positive, HIV positive* They said they wanted to treat the HPV that was on the cervix…To fight the disease… The lesions come about because of HPV, so the thermal ablation treats the lesions. (P0629) *HPV positive, VIA positive, HIV positive*	

### Understanding VIA triage and thermal ablation treatment

Participants reported understanding that the purpose of VIA was to examine the cervix for “damage” caused by the virus or “lesions” that need treatment (**Table 4**). Even those who were HPV-negative and did not undergo VIA described VIA triage as a “confirmatory test,” to see if one has the disease or not. However, there was confusion in Group 2 about the significance of a positive screening test (HPV) with VIA-negative result. One participant expressed concern that the HPV will develop again if not treated, and others felt they still needed some kind of treatment for a positive HPV result, despite the VIA-negative result. One participant reported that the vinegar used in VIA was used to kill the HPV. The majority of Group 2 however viewed a negative VIA triage as an overall negative screening result and expressed relief.

All Group 3 participants understood that an HPV-positive/VIA-positive result meant that treatment was indicated. Many reported initial concerns when lesions were seen on exam, however, they also reported being counseled to not worry, as treatment was available right away (**Table 5**). Participants in this group predominantly expressed acceptance of the results and gratitude that they could proceed with immediate treatment. Participants who underwent treatment with thermal ablation correctly explained that the purpose was to treat lesions and prevent worsening of disease. Of note, we found no differences in understanding between WHIV and HIV-negative participants (**Table 4**).

### Experiences post-thermal ablation and post screen-and-treat

Those who experienced symptoms after ablation viewed these as minor side effects (**Table 3**). The major post-treatment challenge participants reported was having to explain the need for several weeks of abstinence to their male partners. One participant reported that she did not know how to explain the treatment to her husband, as there was no medication that she could show him and felt the conversation would not go well. Other participants reported they were not able to abstain from sex due to the lack of male partner support. Some participants desired male partner counseling to be incorporated in the screen-and-treat program so that their husbands could understand and be supportive of abstinence recommendations. Some felt that same-day screen-and-treat did not allow them the opportunity to discuss treatment with their husbands before receiving it.

Many participants reported that after screening, they shared their experiences with others in their communities, including family, friends, and male partners (**Table 3**). Several participants reported answering questions from other women about screening and addressing misconceptions that kept women from presenting for CxCa screening. Almost all participants felt it was important to share their own experiences with the community and encourage screening uptake (**Table 5**).

## Discussion

The 25 participants of this study, including both WHIV and HIV-uninfected women, exhibited a higher baseline knowledge of CxCa and CxCa screening than reported in prior studies (18). Despite the introduction of several new factors and concept in our study’s screening algorithm – including HPV test, self-collection, a triage step and same-day treatment – participants reported accurate understanding of each step and an overall positive experience. The process of self-collection was highly acceptable, and participants demonstrated understanding and trust towards the HPV test result. Confusion did occur when the VIA result was discordant with the HPV result, indicating the need for additional targeted counseling in this group. Participants demonstrated confidence in their knowledge and expressed desire to share these experiences with their community so that other women are encouraged to attend screening. Counseling, and in particular anticipatory guidance, was key in helping participants understand complex screening procedures and results (**Table 5**).

### Understanding of CxCa and CxCa prevention

Participants demonstrated a higher baseline knowledge of CxCa (e.g. association with sexual transmission, gynecological symptoms, infertility after treatment) and screening awareness (i.e. majority have heard of screening) than previously seen in Lilongwe district (19). This likely was due to recruitment of participants from clinics where women are already engaged in health care, compared to rural populations reached by mobile campaigns (20).

Notably, study participants displayed an understanding of the importance of screening for prevention. While fatalistic views of CxCa still existed (19, 21, 22), they were associated more with late diagnosis. In this study, screening was generally viewed positively and recognized as the way to find disease at a treatable stage and prevent fatal disease progression. Recent studies among women in southern Malawi also captured similar sentiment around the importance of early detection in CxCa screening (23). However, despite adequate knowledge of the disease and understanding of prevention, fears around pelvic exams (e.g. pain and embarrassment) hindered women from previously seeking out screening services; similar acceptability-related barriers have been identified in other studies from low- and middle-income countries (18).

### Self-collection was well accepted

Self-collection of vaginal samples for HPV testing was well-received and valued among our participants for ease of collection, avoidance of embarrassment or discomfort from speculum exams, and quick turnaround time of results. Many felt that self-collection could mitigate the fear and stigma surrounding speculum exams. These findings are consistent with existing literature over the last couple of decades that has shown self-collection to be acceptable across a variety of geographic locations, cultures and age groups (8, 9). Studies specific to African populations showed that women are willing to collect their own samples and that it is a more socially acceptable and feasible method than pelvic exam-based screening methods, such as Pap smear or VIA (24, 25).

Major concerns surrounding self-collection were worry about not collecting it correctly, fear of receiving a positive result, and fear that screening would reveal cancer. These are also well-documented concerns in studies on self-collection HPV testing (26, 27) and have been identified as barriers to screening (28). Factors mitigating these concerns, reported by our participants, were anticipatory guidance of what came next after each step of the screening process, quick results, and availability of same-day treatment. Knowing what to expect with a positive result, and knowing there is treatment right away if the result was positive, were identified as two reassuring factors by participants.

### Understanding of HPV-based multi-step CxCa screening

VIA has been the primary screening method, followed by cryotherapy in Malawi’s screen-and-treat national strategy for CxCa prevention (12). In our study screening algorithm, several concepts including HPV screening, self-collection of vaginal samples, and thermal ablation treatment were new. Additionally, none of the participants (except one) had heard of HPV or its association with CxCa. A large part of the initial recruitment and consent process for the parent study was spent on explaining the relationship between HPV, cervical dysplasia, and CxCa. When this understanding was assessed though IDIs weeks or even months after screening, most participants accurately described that HPV is a virus that causes cervical lesions, and if untreated, can lead to CxCa. This likely is evidence of successful pre-enrollment education, but also suggests that perhaps participants were already familiar with the concept of a virus causing significant illness (e.g., HIV). Exposure to HIV knowledge could have helped make new disease concepts more understandable. Participants engaged in HIV care may have greater knowledge in this area, but in our study, both WHIV and HIV-negative participants displayed good understanding of HPV (**Table 4**).

HPV testing has been shown to be difficult to comprehend for women more accustomed to pelvic exams for screening, with the main challenge being failure to understand why a positive HPV test may not lead to immediate treatment (14, 15). We found similar sentiments among three (of twelve) HPV-positive/VIA-negative participants (**Table 4**). Thematic saturation was reached later with this group due to the variability in understanding. A major hinderance in understanding HPV screening results identified in literature is that patients do not feel that healthcare professionals provide sufficient or understandable information during results delivery (14). When confusion was identified through IDIs, the clinic study staff increased anticipatory guidance of what to except with the VIA triage step and emphasized counseling for those with discordant results so that patients understood why they did not need treatment. The IDIs that occurred after increased counseling efforts revealed less confusion among participants (data not shown).

### Counseling was key to an overall positive screening experience

Ongoing counseling throughout the screening process and anticipatory guidance on what to expect with varying outcomes were key in creating a positive screening experience. Proper counseling leads to understanding, which can increase acceptability, uptake and adherence to routine screening. Counseling can also lead to trust with the healthcare team and mitigates negative psychological effects such as worry and fear, which are well identified barriers to accessing preventative care (29).

Anticipatory guidance was especially helpful in this multi-step screening process. Participants specifically described how knowing what to expect at each step of screening and anticipating what came next after each outcome was helpful in minimizing confusion and reducing negative emotions (**Table 5**). Anticipatory guidance is most practiced in pediatrics to prepare parents on the expected growth and development of their children (30), but not readily employed in cancer screening. Anticipatory guidance is also naturally present through the informed consent process of research, where each step of the study after each potential outcome is clearly reviewed, and the understanding of participants is assessed. While time can be a limitation in real-world cancer screening programs, our study suggests that the time spent on anticipatory guidance, especially if it involves a multi-step screening process, is invaluable for women to prepare for the emotional challenges of CxCa screening.

### Thermal ablation experience

Thermal ablation was well tolerated by the six participants who underwent treatment. Consistent with prior studies, post-treatment side-effects were minimal and most expressed gratitude for being able to receive treatment right away (13, 31). The major challenge reported was difficulty communicating with their male partners at home. Participants felt that because a period of sexual abstinence was recommended post-treatment, they had to inform their male partners of the procedure, which could lead to misunderstandings, conflicts, and for many, inability to comply with post-treatment abstinence. We saw similar challenges in our prior VIA and thermal ablation study (32) that identified male partners as a barrier to returning for follow-up. However, the prior study also identified male partners as a potential source of support in CxCa screening. Including male partners counseling in screen-and-treat services may be vital for women’s safety and acceptance, especially when treatment is indicated.

### Strengths and limitations

This study was successful in reaching and unscreened women. However, being facility-based resulted in a selection of women with access to care. Familiarity with the health system and exposure to health concepts can favorably skew acceptability of the screening procedures. Perspectives from women in rural, hard-to-reach areas need to be considered for successful expansion of this strategy. Notably, a recent study in Malawi that implemented community-based self-collection for HPV screening found that it increased screening uptake (compared to facility-based screening alone) and was acceptable to both clients and health workers (33–35). A strength of this study is that we included both WHIV and HIV-uninfected women and found no differences in their ability to understand results or perspectives on screening steps. Timing of our IDIs weeks to months after screening allowed us to cover topics such as how women had shared their experiences with their community and barriers faced in remaining abstinent for the recommended period following thermal ablation. On the other hand, the delay between the experience and the IDI could have resulted in some recall bias.

HPV-based screen-and-treat experiences through a research study may differ from real-world clinical settings where providers have higher volumes of patients with more limited staffing and resources. For example, good counseling was overwhelmingly identified as being valued by participants in our study, but time may be more constrained in actual clinical practice, limiting provider time to thoroughly counsel patients about potentially confusing results. In addition, our study utilized additional procedures that would not be routinely implemented in non-study settings, such as colposcopy, Pap smear, and/or biopsy, which could have confounded participant’s experiences. However, most patients did not mention the additional procedures, and it did not seem to negatively affect their experiences.

## Conclusion

Self-collection HPV primary screening is a promising method to expand CxCa screening access and increase cervical precancer detection (5, 6). However, the lower specificity of the HPV assay requires a triage test for HPV-positive results to prevent overtreatment (36). As HPV-based multi-step screening is being implemented, our findings provide rich insight for healthcare providers and policymakers surrounding women’s experiences undergoing a same-day HPV-based screen-triage-treat algorithm. We captured what was important to women: convenience of self-collection, quick result turnaround, availability of same-day treatment, and thorough counseling. HPV self-collection can overcome barriers to pelvic exams including discomfort, embarrassment and need for trained providers. While a multi-step screening process with VIA triage can lead to confusion about required follow-up procedures, proper counseling and anticipatory guidance can improve understanding. Incorporating thorough counseling in CxCa screening programs can change women’s perspective of screening, build trust with healthcare systems, and influence healthcare seeking behavior towards routine screening and prevention.

## Data availability statement

The raw data supporting the conclusions of this article will be made available by the authors, without undue reservation.

## Ethics statement

The studies involving humans were approved by Malawi National Health Sciences Research Committee, University of North Carolina at Chapel Hill Institution Review Board. The studies were conducted in accordance with the local legislation and institutional requirements. The participants provided their written informed consent to participate in this study.

## Author contributions

FL: Data curation, Formal analysis, Investigation, Methodology, Project administration, Validation, Writing – original draft, Writing – review & editing. SM: Conceptualization, Data curation, Formal analysis, Investigation, Methodology, Project administration, Writing – original draft, Writing – review & editing. JC: Data curation, Formal analysis, Investigation, Writing – original draft, Writing – review & editing. WD: Data curation, Investigation, Writing – review & editing. JT: Conceptualization, Data curation, Formal analysis, Funding acquisition, Investigation, Methodology, Project administration, Supervision, Writing – original draft, Writing – review & editing. MN: Project administration, Writing – review & editing. LM: Project administration, Writing – review & editing. VM: Supervision, Writing – review & editing. JS: Conceptualization, Data curation, Formal analysis, Funding acquisition, Investigation, Methodology, Supervision, Validation, Writing – original draft, Writing – review & editing. LC: Conceptualization, Data curation, Formal analysis, Funding acquisition, Investigation, Methodology, Project administration, Supervision, Writing – original draft, Writing – review & editing.
